# Effects of Omega-3 Fatty Acid in Nonalcoholic Fatty Liver Disease: A Meta-Analysis

**DOI:** 10.1155/2016/1459790

**Published:** 2016-08-29

**Authors:** Wenxia Lu, Sainan Li, Jingjing Li, Jianrong Wang, Rong Zhang, Yuqing Zhou, Qin Yin, Yuanyuan Zheng, Fan Wang, Yujing Xia, Kan Chen, Tong Liu, Jie Lu, Yingqun Zhou, Chuanyong Guo

**Affiliations:** ^1^Department of Gastroenterology, Shanghai Tenth People's Hospital, Tongji University School of Medicine, Shanghai 200072, China; ^2^The First Clinical Medical College, Nanjing Medical University, Nanjing 210029, China; ^3^The First Affiliated Hospital of Soochow University, Suzhou 215006, China

## Abstract

A meta-analysis was conducted to assess the effect of omega-3 fatty acid supplementation (n-3 PUFAs) in lowering liver fat, liver enzyme (alanine aminotransferase (ALT), aspartate aminotransferase (AST), and gamma-glutamyltransferase (GGT) levels), and blood lipids (triglyceride (TG), total cholesterol (TC), high density lipoprotein (HDL), and low density lipoprotein (LDL)) in patients with nonalcoholic fatty liver disease (NAFLD) or nonalcoholic steatohepatitis (NASH).* Methods.* MEDLINE/PubMed, EMBASE, the Cochrane Central Register of Controlled Trials, CINAHL, Science Citation Index (ISI Web of Science), Chinese Biomedical Literature Database (CBM), and Chinese National Knowledge Infrastructure (CNKI) were searched for relevant randomized controlled trials on the effects of n-3 polyunsaturated fatty acids (PUFAs) in patients with NAFLD from inception to May 2015. Ten studies were included in this meta-analysis.* Results.* 577 cases of NAFLD/NASH in ten randomized controlled trials (RCTs) were included. The results of the meta-analysis showed that benefit changes in liver fat favored PUFA treatment, and it was also beneficial for GGT, but it was not significant on ALT, AST, TC, and LDL.* Conclusions.* In this meta-analysis, omega-3 PUFAs improved liver fat, GGT, TG, and HDL in patients with NAFLD/NASH. Therefore, n-3 PUFAs may be a new treatment option for NAFLD.

## 1. Introduction

Nonalcoholic fatty liver disease (NAFLD) involves the excess accumulation of hepatic fat in the absence of alcohol consumption and is defined by the presence of steatosis (characterized by lipid droplets) in more than 5% of hepatocytes [1]. The histological pattern of NAFLD can progress to nonalcoholic steatohepatitis (NASH). NAFLD is now one of the most common liver diseases worldwide. In Western countries and some regions of China, the prevalence of NASH and NAFLD is 1–5% and 15–39%, respectively [2]. One-third of NASH patients have advanced fibrosis and 20% develop cirrhosis [3]. The pathogenesis of NAFLD is multifactorial and includes excessive inappropriate dietary fat intake combined with peripheral insulin resistance, oxidative stress, and innate immunity [4]. It is frequently associated with obesity, type 2 diabetes (T2DM), dyslipidemia, metabolic syndrome, and cardiovascular disease [5–10].

Currently, several therapeutic approaches for NASH have been proposed. According to EASL-EASD-EASO guideline [11], patients without NASH or fibrosis should only receive counselling for healthy diet and physical activity and no pharmacotherapy for their liver condition and in overweight/obese NAFLD, a 7–10% weight loss is the target of most lifestyle interventions and results in improvement of liver enzymes and histology. No drug has currently been tested in phase III trials and is approved for NASH by regulatory agencies. The drugs studied in trials included insulin sensitizers (metformin [12], thiazolidinediones [13]), antioxidants (vitamin E [14], ursodeoxycholic acid), and cytoprotective and lipid lowering agents (n-3 polyunsaturated fatty acids). However, no specific therapy can be firmly recommended and any drug treatment would be off-label [15–17]. NASH patients with liver failure and/or HCC are candidates for liver transplantation [11].

NAFLD is considered to be associated with an excess of n-6 and a deficiency of n-3 polyunsaturated fatty acids (PUFAs) in the diet [18, 19]. Studies have indicated a lower PUFA content and a higher n-6/n-3 ratio in NAFLD patients [20, 21]. N-3 PUFAs are negative regulators of hepatic lipogenesis and the inflammatory response in mice [22, 23] and have a beneficial impact on hypertension, hyperlipidemia, endothelial dysfunction, and cardiovascular disease [24]. In the present study, we aimed to assess the effect of n-3 PUFAs supplementation in lowering liver fat, liver enzyme (alanine aminotransferase (ALT), aspartate aminotransferase (AST), and gamma-glutamyltransferase (GGT) levels), and blood lipid levels (TC, TG, HDL, and LDL) in patients with NAFLD.

## 2. Materials and Methods

### 2.1. Search Strategy

We searched MEDLINE/PubMed, EMBASE, the Cochrane Central Register of Controlled Trials, CINAHL, Science Citation Index (ISI Web of Science), Chinese Biomedical Literature Database (CBM), and Chinese National Knowledge Infrastructure (CNKI) from inception to May 2015 with no language restriction  [25, 26]. The search terms included were as follows: (NASH or NAFLD or nonalcoholic steatohepatitis or nonalcoholic fatty liver disease or fatty liver or steatosis) and (n-3 PUFA or omega-3 fatty acid or fish oil or n-3 polyunsaturated fatty acid or eicosapentenoic acid or EPA or docosahexenoic acid or DHA) and (Fatty Liver [MeSH]) AND (n-3 polyunsaturated fatty acid) [MeSH]. We also searched the reference lists of each selected study by hand.

### 2.2. Inclusion and Exclusion Criteria

Articles were suitable if the following criteria were satisfied: (i) study design: RCT with the therapy of omega-3 fatty acid, and (ii) study population: patients with NAFLD identified according to the criteria as follows: (1) ultrasonography demonstrating fatty liver and (2) having no secondary hepatic fat accumulation such as significant alcohol consumption, use of steatogenic medication, or hereditary disorders. Studies were excluded for the following reasons: (i) trials that did not provide original data from which sensitivity or specificity could be calculated accurately, (ii) abstracts, letters, leading articles, animal experiments, expert opinion, book sections, case reports, and trials that lacked a control group, and (iii) other causes of hepatic steatosis or steatofibrosis, such as viral hepatitis, autoimmune hepatitis, liver decompensation, or malignancy.

### 2.3. Data Extraction

The search was conducted by two researchers (Wenxia Lu and Jianrong Wang) who read the titles and abstracts of studies independently and eliminated those which did not meet the inclusion criteria. The full texts of articles potentially meeting the inclusion criteria were cross-checked. Included data were extracted by two authors, respectively. The content of the data extracted was decided by discussion before data extraction. In order to avoid subjective bias, the author name, journal name, year, and country were hidden during data extraction. The following data were extracted by two researchers independently: (1) name of the first author, number of patients, year of publication, duration of treatment, daily dose of oral therapy, symptoms, and adverse events; (2) liver biochemistry (plasma ALT, AST, and GGT levels) and blood lipid (TG, TC, HDL, and LDL); (3) liver fatness quantified by needle biopsy and histological assessment, ultrasonography, or inferred by proton magnetic resonance spectroscopy (MRS).

### 2.4. Methodological Quality

The included RCTs were scored with the Jadad composite scale as follows.

*Criteria Used to Grade the Quality of RCTs: The Jadad Scores.* Each study was given one point for each “yes” and 0 points for each “no” in response to each of the following questions:

1. Was the study described as randomized using the words “randomly,” “random,” or “randomization”?
  - An additional point was given if the method of randomization was described and was appropriate (e.g., table of random numbers, computer generated).
  - A point was deducted if the method of randomization was inappropriate (e.g., patients allocated alternately, by birth date, or by hospital number).
2. Was the study described as “double blind”?
  - A point was given if the method of blinding was described and it was appropriate (e.g., identical placebo).
  - An additional point was deducted if the method of blinding was inappropriate (e.g., comparing placebo tablet with injection).
3. Was there a description of the patients who withdrew or dropped out?
4. The maximum number of points was 5.

This is a five-point quality scale, with low-quality studies having a score of ≤2 and high-quality studies a score of ≥3. Methodological quality was independently assessed by the two authors of this study. Each study was given an overall score based on the criteria described above, which was then used to rank the studies. Any disagreement was resolved by consensus.

### 2.5. Data Synthesis

Analyses were conducted using RevMan 5.3. The odds ratio (OR) was presented with its 95% confidence interval (95% CI) only for liver fatness improvement event. Other curative effect evaluation indices were continuous variables, and the random effects model was used to pool the SMD and 95% CI across the included studies. *χ* ^2^ and *I* ^2^ test statistics were used to assess heterogeneity across the studies. When significant heterogeneity was observed (*P* value of <0.1 or *I* ^2^ value of >50%), we analyzed the data using the random effects model. Otherwise, the fixed effects model was adopted. We performed eight analyses to compare the effect of (i) PUFA versus control on ALT change, (ii) PUFA versus control on AST change, (iii) PUFA versus control on GGT change, (iv) PUFA versus control on TG change, (v) PUFA versus control on TC change, (vi) PUFA versus control on HDL change, (vii) PUFA versus control on LDL change, and (viii) PUFA versus control on liver fatness change. We also constructed funnel plots graph to evaluate the presence of publication bias.

## 3. Results

### 3.1. Study Selection and Characteristics of the Studies Included

**Figure 1:**
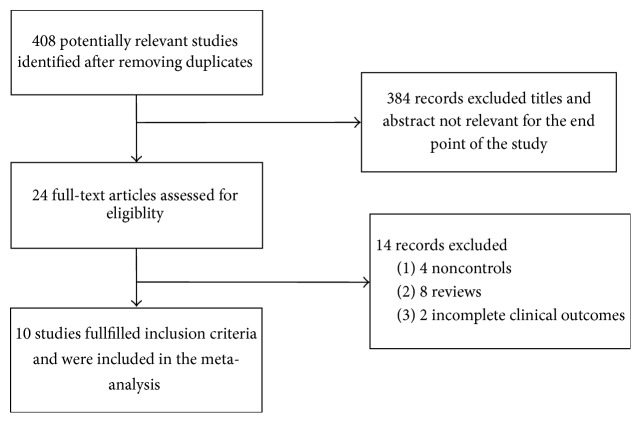
Flow diagram of the studies included in the meta-analysis.

From 408 studies, we finally selected ten RCTs (Figure 1). Data on 577 individuals who participated in the RCTs were analyzed.  Table 1 shows specific information on study design, sample size, intervention, control method, treatment dose, and duration of treatment. All studies were published as full-text articles. Eight studies used placebo as a control and two studies used no placebo or no treatment as a control. The median duration of treatment with omega-3 fatty acids was 12 months (range: 2 months to 18 months). The median dose of PUFAs was 2.85 g/day (range: 0.83–9 g/day) and there were no reports of adverse effects of omega-3 PUFA supplementation in the study reviewed.

Measurement methods used to quantify change in liver fatness included ultrasound (five studies), magnetic resonance spectroscopy (three studies), and liver biopsy (four studies). For the purpose of data pooling and analysis, the “high dose” group was selected as the treatment group for analysis in the study by Chen et al. [35] and Scorletti et al. [28].

### 3.2. Quality Evaluation of the Studies Included

Methodological quality scores ranged from 3 to 5 (Table 2). Eight of the ten randomized studies adequately described the way in which they were randomized. All studies used a double-blinded method, and seven provided specific descriptions of the blinding used. Eight studies described withdrawals and lost cases. Overall, the Jadad scores of all the RCTs were ≥3 points and were thus considered high-quality research.

### 3.3. Meta-Analysis

#### 3.3.1. Effect of Omega-3 Fatty Acid Therapy on Liver Fat

**Figure 2:**
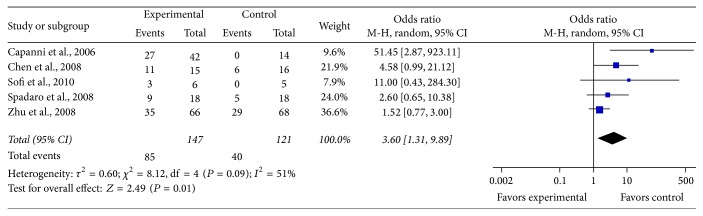
Effects of n-3 PUFAs versus control in liver fat in patients with NAFLD.

Five studies demonstrated fatty liver with ultrasonography. Significant heterogeneity among studies was observed (*χ* ^2^ = 8.12, *P* = 0.09, *I* ^2^ = 51%), with a random effect model, There was a significant pooled OR for the efficacy of PUFA therapy on liver fat (OR = 3.60, 95% CI: 1.31 to 9.89, *P* = 0.01) (Figure 2).

#### 3.3.2. Effect of Omega-3 Fatty Acid Therapy on Liver Function

**Figure 3:**
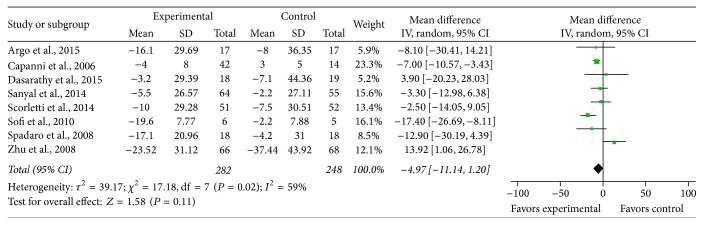
Effects of n-3 PUFAs versus control on ALT in patients with NAFLD.

*ALT*. Eight studies provided sufficient data to enable the calculation of MD and 95% CI for ALT. There was significant heterogeneity between the studies on the effects of PUFA supplementation on ALT (*χ* ^2^ = 17.18, *P* = 0.02, *I* ^2^ = 59%), with the random effects model, and the pooled MD for ALT showed a trend toward PUFA therapy versus control on ALT but did not reach statistical significance (MD = −4.97, 95% CI: −11.14 to 1.20, *P* = 0.11) (Figure 3).

**Figure 4:**
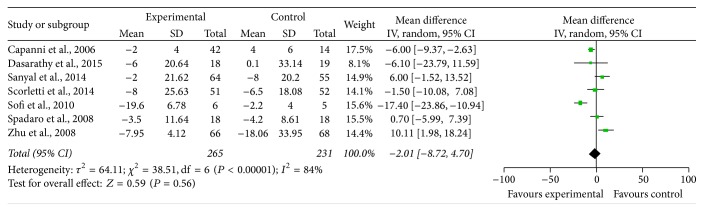
Effects of n-3 PUFAs versus control on AST in patients with NAFLD.

*AST*. Seven studies assessed the effect of n-3 PUFAs on the level of serum AST. Significant heterogeneity was found to exist between the studies on the effects of PUFA supplementation on AST (*χ* ^2^ = 38.51, *P* < 0.00001, *I* ^2^ = 84%). With the random effects model, it was not significant (MD = −2.01, 95% CI: −8.72 to 4.70, *P* = 0.58) (Figure 4).

**Figure 5:**
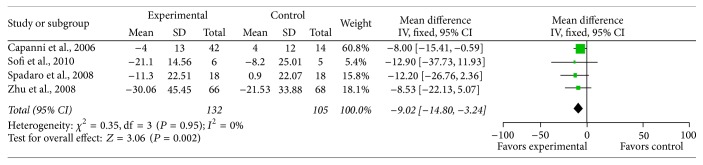
Effects of n-3 PUFAs versus control on GGT in patients with NAFLD.

*GGT*. Four studies reported the effect of n-3 PUFAs on serum GGT reduction, and there was no significant heterogeneity between the studies on the effect of PUFA supplementation on GGT (*χ* ^2^ = 0.35, *P* = 0.95, *I* ^2^ = 0%). Using the fixed effects model, there was significant pooled MD favoring PUFA therapy versus control on GGT (MD = −9.02, 95% CI: −14.80 to −3.24, *P* = 0.002) (Figure 5).

#### 3.3.3. Effect of Omega-3 Fatty Acid Therapy on Blood Lipids

**Figure 6:**
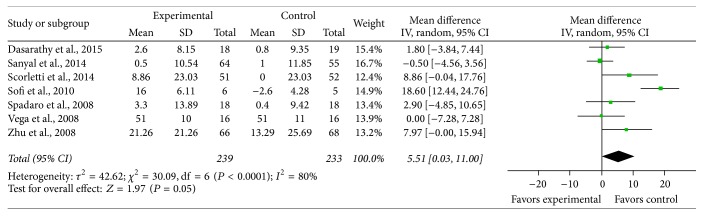
Effects of n-3 PUFAs versus control on HDL in patients with NAFLD.

*Triglyceride*. Nine studies provided sufficient data on triglyceride, and there was significant heterogeneity between the studies on the effect of PUFA supplementation on triglyceride (*χ* ^2^ = 29.17, *P* = 0.0003, *I* ^2^ = 73%). With the random effects model, there was statistical significance between the studies (MD = −35.55, 95% CI: −53.90 to 17.19, *P* = 0.0001) (Figure 6).

**Figure 7:**
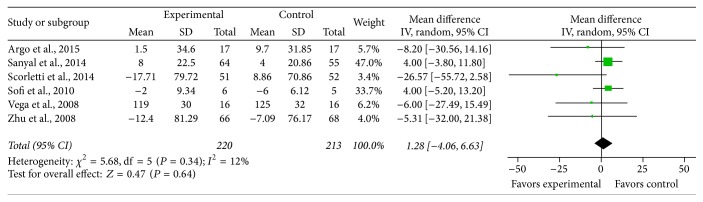
Effects of n-3 PUFAs versus control on LDL in patients with NAFLD.

*Total Cholesterol*. Seven studies provided sufficient data on total cholesterol, and significant heterogeneity was found to exist between the studies on the effect of PUFA supplementation on total cholesterol (*χ* ^2^ = 10.53, *P* = 0.10, *I* ^2^ = 43%). Using the random effects model, it did not reach statistical significance (MD = −10.53, 95% CI: −10.4 to 3.09, *P* = 0.08) (Figure 7).

**Figure 8:**
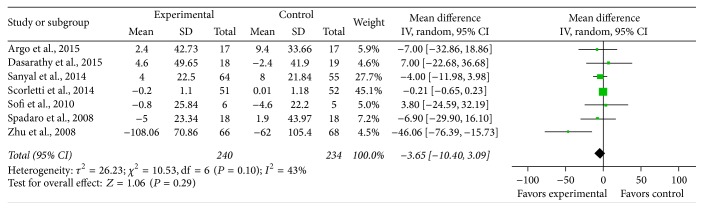
Effects of n-3 PUFAs versus control on TC in patients with NAFLD.

*HDL*. Seven studies reported the effect of n-3 PUFAs on serum HDL reduction, and there was significant heterogeneity between the studies on the effect of PUFA supplementation on HDL (*χ* ^2^ = 30.09, *P* < 0.0001, *I* ^2^ = 80%). Using the random effects model, there was significant pooled MD favoring PUFA therapy versus control on HDL (MD = 5.51, 95% CI: 0.03 to 11, *P* = 0.05) (Figure 8).

**Figure 9:**
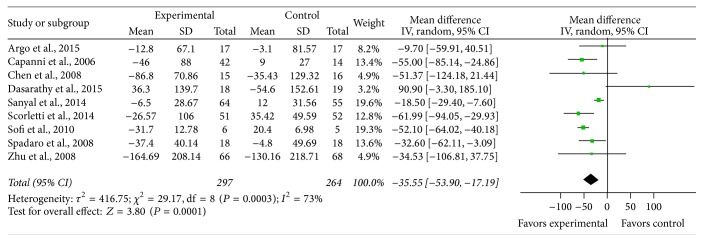
Effects of n-3 PUFAs versus control on TG in patients with NAFLD.

*LDL*. Six studies provided sufficient data on total LDL, and low heterogeneity was found to exist between the studies on the effect of PUFA supplementation on LDL (*χ* ^2^ = 1.28, *P* = 0.34, *I* ^2^ = 12%). With the fixed effects model, it did not reach statistical significance (MD = 1.28, 95% CI: −4.06 to 6.63, *P* = 0.64) (Figure 9).

### 3.4. Publication Bias

**Figure 10:**
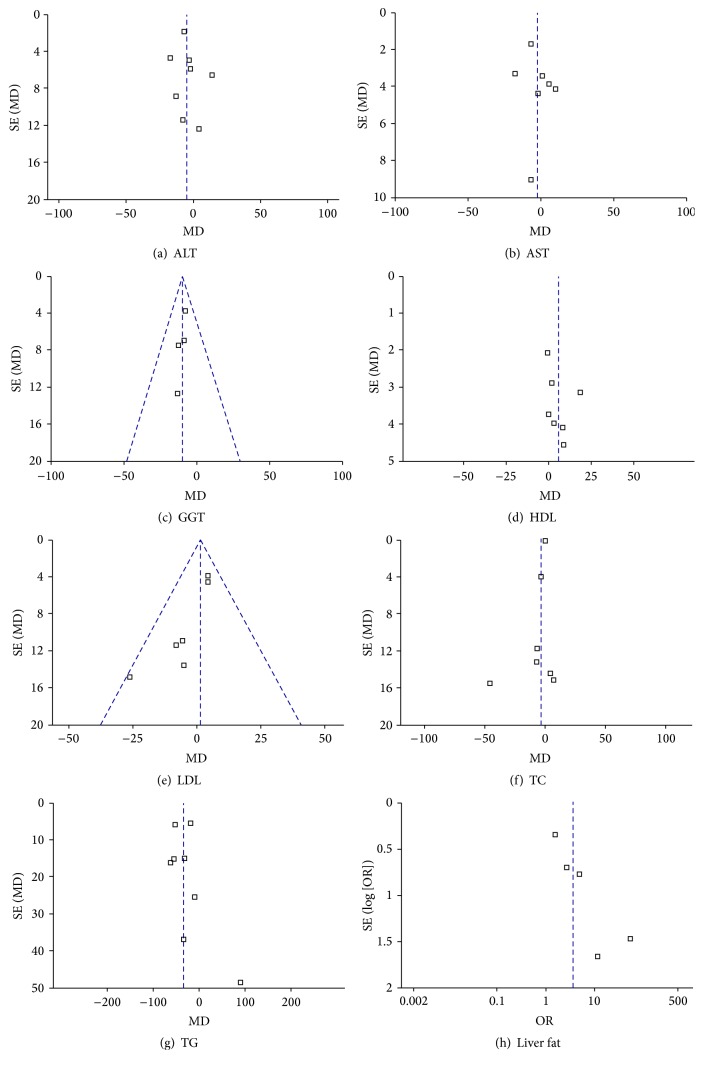
Funnel plots for the meta-analysis.

Figure 10 shows the funnel plots of the meta-analysis. The funnel plot analyses of AST, ALT, GGT, TC, TG, HDL, and LDL showed slight asymmetry, indicating that there was a certain publication bias.

## 4. Discussion

Due to improvements in living standards, changes in lifestyle and the prevalence of obesity, diabetes, and the metabolic syndrome, the annual increase in the incidence of NAFLD has become a global public health problem. Currently, NASH is rapidly increasing as a cause of end-stage liver disease and hepatic carcinoma. At present, there is no registered drug for the treatment of NAFLD, and there is a need to improve therapeutics for this condition. The n-3 PUFAs have been shown to reduce inflammation, enhance insulin sensitivity, and improve hypertriglyceridemia [36]. A meta-analysis indicated that n-3 PUFA supplements significantly decreased the amount of liver fat observed on ultrasound [37]. n-3 PUFAs have also been used to effectively improve dyslipidemia [33, 36, 38]. The present meta-analysis aimed to assess the effect of n-3 PUFAs on liver fat (demonstrated with ultrasonography), liver enzyme levels (ALT, AST, and GGT) and blood lipid levels (TG, TC, HDL, and LDL) in patients with NAFLD and NASH. The results indicated that n-3 PUFAs can optimize liver fat, GGT, TG, and HDL levels in patients with NAFLD, suggesting the therapeutic potential of n-3 PUFAs in this liver disease.

Our results were in accordance with a recent review by Parker et al. [37], which provide a meta-analysis of liver fat, ALT, and AST data from seven RCTs at that time. It showed a benefit on liver fatness and found no significant benefit on ALT and AST levels. The current data also suggest that GGT is affected by n-3 PUFAs. Furthermore, it is well acknowledged that there is high intraindividual variability in liver tests which may reduce the ability to detect significant changes in these parameters.

Although six studies were identified that examined the effect of dietary omega-3 PUFA supplementation on liver fat by ultrasonography, one study could not be included in liver fat analyses because of insufficient data. Two, three, six, three, four, three, and two studies had insufficient data for inclusion in ALT analyses, AST analyses, GGT analyses, TC analyses, TG analyses, HDL analyses, and LDL analyses, respectively.

N-3 PUFA supplements to decrease plasma TG may be associated with glycemic control, as shown in a study of NASH patients with diabetes [31]. It has been demonstrated that n-3 PUFAs activate the peroxisome proliferator-activated receptor (PPAR) alpha, which in turn stimulates fatty acid oxidation [39], and PPAR gamma increases insulin sensitivity [40], inhibits hepatic lipogenesis, and reduces hepatic reactive oxygen species [41]. Besides, patients with NAFLD have been shown to have a greater deficiency of n-3 PUFAs in the diet than healthy controls [42, 43], and a higher n-6/n-3 ratio in NAFLD patients increased lipogenesis leading to steatosis [44].

There were several limitations in our study. First, the number of studies included in this analysis was small. Second, the diagnosis of NAFLD/NASH in the present study was confirmed by liver biopsy, ultrasonography, or MRI. Although ultrasonography is reasonably accurate, it cannot identify fatty infiltration of the liver below a threshold of 30% [37]. Unfortunately, only 4 studies provided posttreatment histology results. Third, a pathogenesis of NAFLD should be established and improved in the near future, to facilitate research into the molecular markers, diagnosis of NAFLD, and target therapies [45–47].

In summary, the results of our meta-analysis support the beneficial effect of n-3 PUFAs in optimizing liver fat, liver enzyme levels (GGT), and blood lipid levels (TG, HDL) in patients with NAFLD and we guess n-3 PUFAs may slow down the progress of NAFLD. More studies with a rigorous design, large sample size, and multiregional cooperation are necessary to examine the therapeutic effect of n-3 PUFA supplementation.

## Figures and Tables

**Table 1:** Baseline characteristics of the included studies.

Authors, year [ref.]	Total	Population	Mean BMI category	Dose n-3/day	Duration	Control	Component n-3/1000 mg capsule	Diagosis
Argo et al., 2015 [27]	34	NASH	Obese	3000 mg	12 months	Placebo	35% EPA + 250% DHA + 10% other n-3s	Liver biopsy + haematochemical
Scorletti et al., 2014 [28]	103	NAFLD	Obese	4000 mg	15–18 months	Placebo	46% EPA + 38% DHA	Liver biopsy or imaging evidence or features of MetS
Sanyal et al., 2014 [29]	119	NASH/NAFLD	Obese	2700 mg	12 months	Placebo	Highly purified EPA ethyl ester	Liver biopsy + haematochemical
Sofi et al., 2010 [30]	11	NAFLD	Obese	830 mg	12 months	Placebo	56.6% EPA + 28.9% DHA	Ultrasonography + haematochemical
Dasarathy et al., 2015 [31]	37	NASH with diabetes	Obese	3600 mg	12 months	without treatment	60% EPA + 40% DHA	Liver biopsy + haematochemical
Zhu et al., 2008 [2]	134	NAFLD	Obese	2000 mg	24 weeks	Placebo	Seal oils	Ultrasonography + haematochemical
Spadaro et al., 2008 [32]	36	NAFLD	Obese	2000 mg	6 months	Without treatment	Not mentioned	Ultrasonography + haematochemical
Capanni et al., 2006 [33]	56	NAFLD	Obese	1000 mg	12 months	Placebo	37.5% EPA + 62.5% DHA	Ultrasonography + haematochemical
Vega et al., 2008 [34]	16	Subset of DHS cohort	Obese	9000 mg	8 wk	Placebo	51.4% C20:5, 23.9% C22:6	Elevated HTGC (MRS) + average ALT within reference range
Chen et al. 2008 [35]	46	NAFLD	Not specified	5000 mg	24 wk	Placebo	Harp seal oil capsules (not specified)	Elevated LFTs and TGs

**Table 2:** Jadad quality scores of the trials included in the meta-analysis.

Study year	Randomization method	Double blinding	Withdrawals/dropouts	Total
Argo et al., 2015 [27]	2	2	1	5
Scorletti et al., 2014 [28]	2	1	0	3
Sanyal et al., 2014 [29]	2	2	1	5
Sofi et al., 2010 [30]	2	2	1	5
Dasarathy et al., 2015 [31]	1	2	1	4
Zhu et al., 2008 [2]	2	1	0	3
Spadaro et al., 2008 [32]	2	2	1	5
Capanni et al., 2006 [33]	2	2	1	5
Vega et al., 2008 [34]	2	2	1	5
Chen et al., 2008 [35]	1	1	1	3
